# An Exploratory, Qualitative Study of How Organizations Implement the Hierarchy of Controls Applied to *Total Worker Health*^®^

**DOI:** 10.3390/ijerph181910032

**Published:** 2021-09-24

**Authors:** Heidi L. Hudson, Anita L. Schill, Reid Richards

**Affiliations:** 1 Centers for Disease Control and Prevention, National Institute for Occupational Safety and Health, 1150 Tusculum Ave., Cincinnati, OH 45226, USA; 2 Centers for Disease Control and Prevention, National Institute for Occupational Safety and Health, 395 E St. SW, Washington, DC 20201, USA; anita.l.schill@gmail.com; 3 Centers for Disease Control and Prevention, Division of HIV/AIDS Prevention, 8 Corporate Blvd. NE, Atlanta, GA 30329, USA; reidrichards@cdc.gov

**Keywords:** Total Worker Health, hierarchy of controls, qualitative study, workplace safety, implementation science, future of work, occupational safety and health

## Abstract

Understanding of how *Total Worker Health*^®^ (TWH) guidelines are implemented in employment organizations in the USA is not well understood. The purpose of this study is to explore how the principles of the Hierarchy of Controls Applied to NIOSH Total Worker Health (TWH HoC), have been implemented among organizations featured as Promising Practices for TWH between 2012–2019, with special focus on the work-related issues of fatigue, stress, sedentary work, and tobacco control. We also sought to identify benefits, obstacles, and lessons learned in the implementation of the TWH HoC. Eighteen organizations were identified to be included in the study. Using a qualitative cross-sectional design and purposive sampling, seven in-depth interviews were conducted with thirteen key informants. The Consolidated Framework for Implementation Research was used to guide the thematic analysis and interpretation of qualitative data. Four themes identified include recognition of the TWH approach and TWH HoC, implementation of the TWH HoC, barriers and facilitators in addressing specific work-related issues, and implementation climate primes benefits, obstacles, and lessons learned. The inner setting (i.e., culture, implementation climate, readiness for implementation) of organizations was a prominent determinant of the implementation of integrated worker safety, health, and well-being interventions.

## 1. Introduction

**Figure 1:**
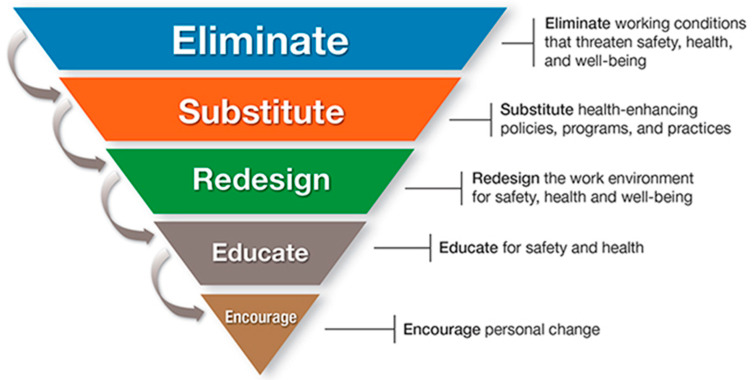
Hierarchy of Controls Applied to NIOSH *Total Worker Health*^®^.

Since the development of the *Total Worker Health*^®^ (TWH) program led by the National Institute for Occupational Safety and Health (NIOSH) in the United States of America (USA), several guidelines have been created to aid employers in the implementation of integrated interventions that collectively address worker safety, health, and well-being [1,2,3]. Among these guidelines, the Hierarchy of Controls Applied to NIOSH Total Worker Health (hereinafter referred to as TWH HoC) was published in Fundamentals of Total Worker Health Approaches [2] as a conceptual model designed to aid employers and other professionals interested in implementing workplace safety and health programs aligned with TWH approaches. The TWH HoC expands the traditional industrial hygiene Hierarchy of Controls [4] and addresses strategies to advance worker well-being. The two Hierarchies of Control are complementary and underscore the foundation of worker safety and health. Figure 1 illustrates the TWH HoC and the five control levels arranged in order of effectiveness (most to least effective order) [5].

Application of the TWH HoC begins with eliminating working conditions that threaten safety, health, and well-being. This level is instituted at the highest tier of the organization and its approaches are often impersonal and unyielding; customization or individual considerations will not be included in elimination controls. Examples of the eliminate control include organizational-wide policies for tobacco-free work environments, organizational and management policies that eliminate root causes of stress, such as excess demands or workplace bullying, and policies that provide workers with increased flexibility and control over their work and schedules. The second level focuses on substitution or replacement of unsafe, unhealthy working conditions or practices with safer, health-enhancing policies, programs, and management practices that improve the culture and safety of the workplace. Examples of substitute controls include replacing food options in workplace common areas with healthier versions, encouraging reports of unsafe work practices without fear of reprisal, and self-funded health insurance. The third level, focused on controls to redesign the work environment for safety, health, and well-being, is characterized by the improvement of work-focused interventions. Examples of redesign include the addition of sit-stand workstations to address sedentary work, improved shift work scheduling, and enhancement of employer-sponsored benefits. The fourth control level, educate for safety and health, focuses on interventions and practices aimed to enhance individual knowledge for all workers. Examples include safe patient handling training, motivational interviewing, peer-led meetings, and telephonic counseling sessions. The fifth level is meant to encourage personal change to improve individual and group health, safety, and well-being. This level emphasizes healthier choice making and may include email and text message prompts, posters, and incentive programs.

Despite development of this hierarchy, an understanding of how national worker safety and health guidelines are adopted and implemented in employment organizations is not well understood. In fact, dissemination and implementation (D&I) science has scantly been applied to the fields of occupational safety and health (OSH), [6,7,8]. D&I science is a multidisciplinary, rapidly emerging field that aims to improve the relevance and uptake of research-based knowledge in real-world settings [9]. Several scholars in the field of OSH recognize there is a need to draw from the field of D&I science to deepen our understanding of factors that influence adoption of evidence into practice as well as further our understanding of how research is applied to policy [8,10,11,12]. Further, scholars recommend using qualitative and mixed methods as they are expected to provide contextual meaning on the effectiveness of interventions [13].

The TWH HoC has been used to inspire occupational health nurses to implement new approaches for workplace safety, health, and well-being [14]. Some researchers have used it as a framework to evaluate and interpret findings on the effectiveness of integrated occupational safety, health, and well-being interventions and determine priorities and design for future interventions [15,16]. Baron and colleagues [17] adapted the TWH HoC to the social ecological framework to demonstrate how employment and work—a major social determinant of health—are impacted by other domains of social influence. Reolofs [18] used it to develop a conceptual model toward protecting employees and promoting their well-being during and after crises such as weather disasters, pandemics, and acts of terrorism.

Despite its use in the literature, it is unclear how the TWH HoC has been implemented within employment organizations. In the face of rapid changes to the workplace, work, and the workforce, such an understanding will help to elucidate how NIOSH TWH guidelines and recommendations are put into practice in organizations facing these emerging pressures. Work of the future will have significant implications for workers, employers, and society as both anticipated and unforeseen issues impact one another and often interrelate to each other, increasing their complexity [19]. Consequently, there is a need for an increased understanding of why and how existing OSH guidance, such as the TWH HoC, is implemented. This understanding will provide insights to help OSH professionals prepare for future opportunities and challenges.

Punnett et al. [10] and Sorensen et al. [20] cite the need for agreement about the necessary components for an effective TWH approach based in both conceptual and practical constructs. Even similar vocabulary or shared understanding of the terms by which implementation of integrated worker safety, health, and well-being programs are described may aid in the advancement of TWH approaches in the field [6]. Characterization of the TWH HoC as a standardized framework and concurrence about its distinct control levels may offer a clear model for workplaces seeking to protect and promote worker safety, health, and well-being. By understanding the factors which influence adoption and implementation of the TWH HoC in real-world settings OSH researchers can elucidate the needs of employers in identifying and mitigating risks posed by work now and in the future [21].

The overall aim of this study is to explore how the principles of the Hierarchy of Controls Applied to NIOSH Total Worker Health have been implemented among organizations featured as Promising Practices for Total Worker Health (hereinafter referred to as Promising Practices) [22] between 2012 and 2019, with a special focus on the work-related issues of fatigue, stress, sedentary work, and tobacco control. The five areas of special emphasis were chosen because, at the time of the study, organizations sought information on these topics from the NIOSH TWH program. Promising Practices is a periodic feature of the NIOSH TWH in Action! quarterly e-newsletter that spotlights a selected organization’s efforts to implement programs, policies, practices that reflect the TWH approach to worker safety, health, and well-being [23].

The primary objectives of this study are:

1. To explore how organizations featured in Promising Practices have implemented the principles of the Hierarchy of Controls Applied to NIOSH Total Worker Health in their programs, policies, and practices that address worker safety, health, and well-being.
2. To explore how organizations featured in Promising Practices have used the TWH approach to address fatigue and sleep, work-related stress, sedentary work, and tobacco control.
3. To identify benefits, obstacles, and lessons learned in the implementation of the TWH HoC among organizations featured in Promising Practices.

## 2. Materials and Methods

### 2.1. Study Design

This exploratory study used a qualitative cross-sectional design involving in-depth interviews to gather information about how organizations implemented or are implementing core principles of an integrated approach toward worker safety, health, and well-being. The Consolidated Framework for Implementation Research (CFIR) [24] serves as the guiding theory for understanding the adoption of TWH HoC. The NIOSH Institutional Review Board Human Research Protection Program determined this study to be exempt from requirements for research involving human subjects.

### 2.2. Study Setting and Sample

The study population was drawn from organizations featured in Promising Practices articles published between 2012 and 2019 [22]. These articles feature early adopter organizations targeting the conditions of work to improve the safety, health, and well-being of workers on and off the job. Early adopter organizations, a commonly used term at NIOSH derived from the Diffusion of Innovations theory [25], will be used in this study to broadly describe organizational change agents that have embraced work-related integrated safety, health, and well-being approaches to advance worker well-being on and off the job. Using a purposive sampling method, a total of 18 articles were selected based on inclusion and exclusion criteria. Inclusion criteria were determined by the research team (HH, AS, RR) through independent article reviews and team deliberations on characterizations and applications of the TWH HoC. To be included in the sample, the article needed to describe a broad application of the TWH HoC and details about the process of implementation in the organization’s TWH-related program. Articles were excluded if they did not discuss aspects of the TWH HoC. Of the 18 articles reviewed, a total of 8 organizations were eligible for the study.

Table 1 provides a description of the organizations in the study sample. Organizations are characterized by size, sector, span of operations, manufacturing or non-manufacturing, and program maturity. The number of key informants that were involved in the organizational interviews is also identified. Six out of the seven organizations had more than 500 employees. Two of the seven organizations were in the government sector and the remaining organizations were in the private sector. There was an even distribution between the span of operations with five of the seven considered as non-manufacturing. Five of the seven organization’s health, wellness, or TWH program had been in place for less than 15 years.

### 2.3. Data Collection Procedures and Measures

The research team consists of current and former core team members of the NIOSH TWH program, including a senior science advisor (AS), a public health analyst (RR), and a health scientist trained in qualitative methods (HH). An interview guide was developed by the research team (AS, HH, RR) and included open-ended questions that were associated with study objectives. Interview questions asked were relevant to how the organizational program evolved since the publication of the Promising Practices article; application of the TWH approach relevant to organizational safety, health, and well-being concerns; examples of each of the levels of controls in the TWH HoC; use of TWH approach to address tobacco control, sedentary work, fatigue, and work-related stress; and experienced organizational benefits, barriers, and lessons learned in using the TWH HoC or TWH approach. The special focus topics of fatigue and sleep, work-related stress, sedentary work, and tobacco control were selected as these topics were most mentioned by organizations seeking information from NIOSH at the time of data collection.

A research team member (HH) contacted representatives from each of the selected organizations via email to enlist key informants in a one-hour in-depth telephone interview. A key informant is an individual with either direct or indirect responsibility for implementing programs, policies, and practices that safeguard the safety, health, and well-being of their organization’s workforce. The key informant may or may not have been involved in development of the Promising Practices article. Once a mutual meeting time was identified, the identified key informant(s) received a letter by e-mail confirming the interview schedule and an overview of the interview focus, including the interview questions, the relevant Promising Practices article, a web link to the Hierarchy of Controls Applied to NIOSH Total Worker Health [5], an article that provided context for the TWH concept and clarity around the TWH HoC [14], and brief biographies of the three interviewers. Providing this information prior to the interview allowed the primary key informant to invite other subject matter experts in their organization to participate in the organizational interview, gather relevant program specifics from other sources and offered the opportunity for the key informants to prepare complete and accurate responses. There was no limit on the number of key informants representing the organization in the interview. All subjects gave their informed consent before participating in the organizational interview. Participants were informed at the time of the interview that they would be given an opportunity to review the manuscript prior to journal submission. After the research team reviewed transcripts and notes, if deemed necessary, a research team member contacted the appropriate key informant to clarify points in the discussion.

Semi-structured interviews were conducted between 16 July 2019 and 09 August 2019, with two team members asking the interview questions (AS and HH) and the other team member serving as notetaker (RR). All interviews were transcribed by a research team member (RR) using MS Word. Of the eight organizations eligible for interviews, seven were scheduled and one was declared lost to follow-up after multiple, failed attempts to reach organizational contacts. After each interview, the notetaker emailed the interview transcription to be reviewed by all members of the research team for accuracy and saved the file in a shared folder. Transcripts were uploaded into Dedoose software (version 8.2.14) for data coding and analysis.

### 2.4. Qualitative Data Analysis

A mixed inductive-deductive approach was used for data coding and analysis. Initially, each member of the research team (AS, RR, HH) independently reviewed the interview transcripts and then discussed preliminary findings that aligned under the research objectives. After a series of joint discussions drawing from the data, scientific literature in TWH, and expertise in TWH, the research team determined that the Consolidated Framework for Implementation Research (CFIR) [24] would be useful for the data analysis and interpretation of the findings. The CFIR was chosen because of its comprehensiveness for understanding determinant factors or potential barriers and facilitators in the implementation of interventions in an organizational context. This is particularly important given the complex interplay of organizational resources needed to assess, plan, and implement the TWH HoC or other TWH-related interventions.

The CFIR draws from multiple disciplines (e.g., psychology, sociology, and organizational change). CFIR includes five domains (i.e., intervention characteristics, outer setting, inner setting, individuals involved, and implementation process) which are primary contextual factors that influence implementation effectiveness. Within each domain is a pragmatic constellation of thirty-nine constructs believed to influence implementation, positively or negatively [24]. The intervention characteristics domain refers to the attributes of an intervention that impact the implementation success and includes its perceived internal or external origin or source, relative advantage, adaptability, trialability, complexity, evidence quality and strength, design quality and presentation, and cost. The outer setting domain refers to the external influences of intervention implementation and includes cosmopolitanism or the level at which the implementing organization is networked with other organizations, peer pressure, and external policies and incentives. The inner setting domain refers to characteristics of the implementing organization. The fourth major domain are the individuals involved and include those who are important to the influence of the intervention, integral to the process of implementation, and may come from the inner setting or outer setting. The fifth domain is implementation process which is an interrelated series of subprocesses that don’t necessarily occur sequentially and may or may not be planned or spontaneous, and linear or non-linear. Appendix A Table A1 describes the CFIR domains and constructs in the context of the TWH HoC and TWH-related approaches.

An initial codebook was developed that applied a priori codes relevant to the study objectives. Data were analyzed between January and April 2021. Using Dedoose qualitative data analysis software, a single coder (HH) applied a priori codes to the transcripts. Emerging and post hoc codes relevant to CFIR were subsequently added. After first round coding was completed, three transcripts were compared between HH and RR to ensure the data coding and interpretation was prudent. Coded data were reviewed and collated into potential themes and reviewed to refine themes. The most salient CFIR domains and constructs that aligned with the study objectives steered the identification and finalization of primary themes and subthemes. Because the CFIR was used for analysis and not part of the data collection instrument, only certain domains and their constructs were relevant to this study. Exemplary quotes were identified that aligned to each theme and subtheme. The Consolidated Criteria for Reporting Qualitative Research was used to structure reporting of the findings [26].

## 3. Findings

We interviewed thirteen key informants from seven employment organizations (Table 1). All key informants had direct or indirect roles responsible for implementing programs, policies, and practices that safeguard the safety, health, and well-being of workers in their organization. The findings from this study are reported within four prominent themes: recognition of the TWH approach and TWH HoC, implementation of the TWH HoC, barriers and facilitators in addressing specific work-related issues, and implementation climate primes benefits, obstacles, and lessons learned. The first theme emerged inductively from the data, and the remaining three themes align to the study objectives. The themes, subthemes, and their alignment to relevant CFIR domains are highlighted in Table 2 and subsequently described.

### 3.1. Theme 1: Recognition of the TWH Approach and the TWH HoC

A prominent theme that emerged in the data was recognition of the TWH HoC and of the overall TWH approach among all organizations interviewed. Relevant to the CFIR domains of intervention characteristics and inner setting, this finding speaks to the implementation attributes of intervention source, relative advantage, adaptability, implementation climate, compatibility, and readiness for implementation. These attributes were among the main constructs that emerged when key informants discussed their organization’s overall approach to worker safety, health, and well-being and whether their organization’s interventions were directly influenced by NIOSH TWH-related guidelines.

#### 3.1.1. Subtheme: Varied Awareness of the TWH Approach and the TWH HoC

An inductive subtheme that emerged was the varied awareness of the TWH approach and the TWH HoC. Two organizations indicated they were aware of both the TWH approach and the TWH HoC. One organization indicated they were aware of the TWH approach but not the TWH HoC. One organization indicated they were not aware of the TWH HoC or the TWH approach. Three organizations did not indicate they were aware of the TWH approach or the TWH HoC. This finding is possible because the TWH HoC was published in 2016 and some of the articles were published prior to its release. It is also important to note that organizations selected to be featured as Promising Practices may or may not purposely follow the TWH approach and were recognized based on how they targeted the conditions of work to improve the safety, health, and well-being of workers on- and off-the-job.

“I didn’t know about the hierarchy of controls applied to TWH and am embarrassed. It’s taken a long time, and [it’s] still taking time to see this approach. Not everybody knows about it…You can’t be thinking about overall employee wellness without thinking that employees feel included, well, healthy, and in a physically safe environment. The TWH model is helping me to broaden my perspective in valuing all parts of the employee.”

[Organization A]

#### 3.1.2. Subtheme: The Principles of TWH Are Part of Existing Organizational Values for Building a Healthy Work Culture

Despite the varied awareness of the TWH HoC or the TWH approach, the principles of an approach consistent with TWH align with existing organizational efforts for building and maintaining a safe and healthy work culture. Four organizations inferred that TWH principles were only a part of their organization’s existing approach towards building and maintaining a workforce culture of health. This relates to the compatibility (a construct of inner setting domain) of the TWH approach or the TWH HoC with organizational values, workflows, or systems and the values and meaning attached to involved individuals implementing the intervention; otherwise, relevant to the construct of implementation climate (a construct of the inner setting domain). Also relevant is the intervention source (a construct of the intervention characteristics domain) referring to the perceptions of key stakeholders about whether the motivation for the TWH approach or the TWH HoC was internally or externally developed. Organizations C, D, F, and G discussed how the tenets of TWH (i.e., elimination of occupational hazards, leadership engagement, and worker involvement) were part of how their organizations operate.

“I do remember that it was mentioned in some meetings I took part in…These ideas are so baked into how we conduct our core business. It’s hard to be explicit because it’s already interwoven. I think it was [an] integral part of that process, but not in an overt checklist fashion.”

[Organization G]

#### 3.1.3. Subtheme: The Total Worker Health Approach Leverages with Traditional Occupational Safety and Health Approaches to Address Worker Health More Broadly

When tackling working conditions that threaten the safety, health, and well-being of workers, some organizations discussed how the facets of the traditional HoC were leveraged with the TWH HoC. Respondents often discussed that building off the organization’s environmental health and safety plan was a key strategy for beginning to evaluate work-related health risks and devise solutions to address them. Organizations also referred to their use of multi-level or comprehensive approaches that collectively addressed work-related risks examined in this study (e.g., work-related stress, sedentary work, and sleep and fatigue), other potential hazards (e.g., repetitive motion, slips, trips and falls, chemical exposures) and health-enhancing environments (e.g., access to healthy food). This subtheme refers to the construct of adaptability (a construct of the intervention characteristics domain) of the intervention into the organizations.

“The Total Worker Health approach enabled us to incorporate wellness into the safety, health, and wellness strategy…We needed to have a safety strategy and our office was attuned to this. We incorporated wellness into this national strategy which got a lot of attention. For wellness to be included is quite incredible.”

[Organization A]

### 3.2. Theme 2: Implementation of TWH HoC

Theme two is relevant to study objective one as it focuses on determining factors in the implementation of the TWH HoC among early adopter organizations featured as Promising Practices published by NIOSH. In the context of implementing TWH HoC, the CFIR domains that were most evident when discussing the application of each of the five controls include intervention characteristics and inner setting. Respondents frequently discussed the significance of available resources (a construct of inner setting domain) in the implementation of the TWH HoC. Subthemes are relevant to determining factors (i.e., CFIR constructs) in the implementation of each of the five levels of controls.

#### 3.2.1. Subtheme: The Eliminate Control Was Commonly Used and Trialed among All Organizations

The eliminate control was reported as commonly used by all organizations interviewed. Respondents shared numerous programs, policies, and practices that applied the eliminate control including:

- implementing company-wide tobacco-free policies,
- use of robots in material handling to eliminate occupational exposures to lifting heavy loads and use of awkward postures,
- elimination of sugar-sweetened beverages sold at the workplace,
- elimination of electric cords from floors to remove hazards for slips, trips, and falls,
- reductions in shiftwork rotations and hours to prevent work-related fatigue, and
- use of machine guarding to prevent traumatic injuries.

Relating to the trialability (a construct of the intervention characteristics domain) of the eliminate control, several organizations referred to a stepwise process, involving multi-levels of controls that led to eliminating and reducing working conditions that threaten the safety, health, and well-being of workers. For example, Organizations B, E, and G referred to their efforts for eliminating unhealthy food and beverage options in the workplace through organizational policies and food service contracts.

“[We] are eliminating sugary beverages from all facilities and [sites]. [They are] not an option anymore. [We] put in healthier beverages like flavored waters. We can teach you about how much sugar is in a soda, but now we’re going to make that hazard nonexistent in [the] facility.”

[Organization G]

Concerning the CFIR inner setting domain, nearly all the organizations discussed a cultural perspective that concentrates on eliminating work conditions that could be threatening to employee safety and health, the first level of control.

“When redesigning our headquarters building, a concrete example was our IT department collaborated with safety and health looking at trip hazard reduction. All our technology in conference rooms is now in the ceiling…That was undertaken from the safety committee to manage trip hazards, so that’s an example of the philosophy permeating the organization. There’s collaborating, and value placed to spend money on technology to put cords in the ceiling.”

[Organization B]

#### 3.2.2. Subtheme: Adaptability and Resources Are Important in the Substitution of Unhealthy Working Conditions

In responses for using the substitute control for unhealthy working conditions or practices, most organizations referred to several health enhancing policies, practices, and programs including:

- healthy movement policies,
- access to an onsite psychologist,
- placement of healthier choices in workplace cafeteria, and
- use of a lift-assist device.

Often organizations suggested that substitution controls were adaptable to address threats to worker safety, health, and well-being. They reported the importance of the adaptability of the intervention (a construct of intervention characteristics domain) and dedicated resources (a construct of the inner setting domain) for successful intervention implementation. For instance, Organizations B and D discussed how they reduced worker injury risk by using physical resources (i.e., robots and adjustable furniture) to implement a substitution control.

“The lift assist device is the people-powered version of the robot. During peak times, there are three stations where the individual has a pistol grip handle with air suction called an air assist device. Stick that handle on a box, pick up the box, turn, and place it on the conveyor. That’s a substitute because it still means the person moves the box, but the weight of the box to the individual is no more than 10 lbs.”

[Organization D]

#### 3.2.3. Subtheme: The Redesign Control Was the Most Frequently Used Control and It Was Likely to Provide Both Quality and Advantage

The redesign control was the most widely discussed control among all five controls. Respondents provided several examples of the redesign control, including:

- a worker-tailored pacing program for production,
- health insurance based on salary,
- enhanced lighting for an aging workforce,
- upgraded facility for violence protection, and
- an onsite nap room for workers to re-energize during the workday or de-stress before leaving work.

Within the breadth of examples reported, relative advantage and evidence strength and quality (both constructs of the intervention characteristics domain) were the most relevant for the redesign control. For example, Organizations B and E each described how they have redesigned aspects of their facilities, leadership programs, and health benefit plans to improve the safety, health, and well-being of their workforce.

“[We’ve completed a] redesign [of the] work [environment] for violence prevention. [We] don’t have a higher risk than many other companies but take it seriously. [We] have interview rooms with exterior exits and [the] ability to be locked down, panic alarms, and bulletproof glass…We’ve done physical redesign for violence prevention and response, education with that, and [a] thoughtful approach for eliminating those hazards.”

[Organization B]

“I mentioned our benefit plan being redesigned to value-based insurance design. [We offer] free medications and supplies for diabetics. [We redesigned healthcare] premiums based on salary. [The] health savings account contribution from [the] employer [is] now based on salary—[the] less you earn, [the] more you get from [the] employer.”

[Organization E]

#### 3.2.4. Subtheme: The Education Control Offers Advantage by Coupling with Other Organizational Efforts

The education control was the second most discussed control among organizations interviewed. The education control was often discussed as a method to complement other controls for addressing workplace conditions that threaten the safety, health, and well-being of workers rather than as a method to solely address hazardous working conditions. The combined use of the education control with other organizational efforts that protect worker safety, health, and well-being refers to the control’s relative advantage (a construct of the intervention characteristics domain). Reported programs and practices included:

- training for supervisors and leaders that aims to reduce work-related stress among workers,
- health risk appraisals accompanied by health education,
- tobacco cessation training,
- corporate athlete programs, and
- onsite teaching of yoga and mindfulness meditation retreats for workers.

Organizations A, B, and E discussed supervisory-focused trainings that educate leaders on their influence on worker’s health and safety and empowering leaders to access available resources (a construct of the inner setting domain) for high-risk workplace situations.

“[There has been] a big movement to educate supervisors on their role holistically, not just [the] HR aspects of being a supervisor. Often, people are promoted to supervisory positions and haven’t had experience supervising…That impacts psychological safety and creating [safe] work environments and overall total worker health.”

[Organization A]

#### 3.2.5. Subtheme: An Organizational Culture Built around Healthier Choice Making Underscores the Encourage Control

Organizations provided a myriad of programs and practices to encourage workers to make personal changes to their health, safety, and well-being both on and off the job. These supports included:

- employee recreational equipment to use during breaktime and meetings,
- special interest groups and outdoor clubs,
- encouraging civility in the workplace,
- health coaching offered through employee assistance programs (EAP), and
- periodic health-related workplace campaigns.

Inner setting domain constructs of culture, implementation climate, and readiness for implementation are likely key determinants for the encourage control. Organizations B, C, G, and F discussed the importance of the encourage control and suggested that there can be degrees of organizational influence within the encourage control and healthy choice making can be built within the organizational culture.

“This year on the 75th anniversary of [our company] credo, [we] updated the credo. [It’s] very much a part of who we are. It’s at the front door of every building we operate in and we added our commitment to employee well-being. Safety had been in there, but we added employee health and well-being. It’s a huge statement to have something at that level.”

[Organization G]

### 3.3. Theme 3: Barriers and Facilitators in Addressing Specific Work-Related

The inner setting domain was a notable determining factor when discussing organizational programs, practices, or policies that address the work-related issues of sleep and fatigue, tobacco use, and sedentary work. Among these three issues of concern, each indicated unique inner setting domain constructs. Culture and available resources were highlighted as important inner setting domain constructs for work-related sleep and fatigue, tobacco control, and sedentary work, whereas planning and engaging (constructs of the implementation process domain) were discussed as important factors in the application of programs, policies, and practices that address work-related stress.

#### 3.3.1. Subtheme: Leadership Engagement, Available Resources, and Access to Information Are Possible Facilitators or Barriers for Organizational Efforts That Focus on Work-Related Fatigue and Sleep

The redesign, education, and encourage controls were used to address work-related sleep and fatigue among the organizations. Organizations described their approaches for addressing work-related sleep and fatigue through a lens of injury prevention and worker health. Organizations provided numerous examples of programs, policies, and practices that addressed work-related sleep and fatigue. These included:

- limited work hours during peak seasons,
- access to onsite nap programs and quiet rooms,
- engaging leadership in encouraging frequent rest and stretch breaks,
- peer support,
- worker training and education,
- providing flexible work schedules, and
- offering sleep care benefits through healthcare plans.

Among the CFIR domains, intervention characteristics and inner setting are likely determining factors for implementing work-related sleep and fatigue interventions. Readiness for implementation (an inner setting construct) was key as respondents referred to the importance of leadership engagement, available resources, and access to knowledge and information in their examples of controls that address work-related sleep and fatigue. For example, Organization F discussed their plans for how they were addressing sleep and fatigue related to shiftwork.

“At the beginning of next year, [we are] looking at different dimensions of [fatigue]. Times to start and stop shifts, maximum number of working hours, minimum rest time between shifts, fatigue education and training…Our initial focus was on shift workers, but everyone can be fatigued.”

[Organization F]

The relative advantage and complexity (constructs of the intervention characteristics domain) of eliminating or reducing shift work was addressed by a few of the organizations. Organizations C, D, and F described to how they are redesigning their work environment to reduce fatigue and shiftwork among higher-risk jobs by rotating or reducing staff and hours of shiftwork. Their approaches are combined with policies and onsite resources (i.e., nap room and training) that encourage fatigue management.

“We need a place for someone to come [to sleep], [if for] whatever reason, you don’t sleep and are tired. No matter what you do, you need sleep. [Workers] are sleeping [at work] for 30–40 min. We’re accepting that behavior and condoning it if we do nothing because we know it exists… [we] just implemented [our napping program] this month.”

[Organization C]

Organizations E and G briefly discussed the complexity within their organization for addressing work-related sleep and fatigue.

“There has been work [to address work-related sleep and fatigue], but it’s complicated. [One of our states] has a 2.4% unemployment rate. We’re up against workforce challenges. Some people really prefer the 12 h shift. It gives them additional time ‘off’. It’s an interesting challenge.”

[Organization E]

#### 3.3.2. Subtheme: Culture and Available Resources Were Indicated as Important Supports for Organizational Tobacco Control Efforts

Six of the seven organizations interviewed had tobacco-free policies, addressing the elimination control. Some organizational policies emphasized tobacco-free requirements on all property, including vehicles, and prohibited use of electronic cigarettes. Education and encourage controls were used by most organizations and used in concert among organizations that had organization-wide tobacco-free policies. Culture and available resources (constructs of the inner setting domain) were prominently reported for tobacco control among many of the organizations interviewed. Organizations D, E, F, and G discussed how they encourage a tobacco-free culture of health for workers while they are at work and away from work using multi-level controls.

“Tobacco control [has] a global policy. [We] used to have policies by location, [but] now there’s a global policy with smoke free requirements across all sites. [Our] latest position was to include e-cigarettes in this…Education is always available…[we] subsidize medications for tobacco cessation.”

[Organization F]

#### 3.3.3. Subtheme: Organizational Culture and Available Resources Were Suggested to Provide Multi-Level Efforts for Addressing Sedentary Work

Among all seven organizations interviewed all five levels of controls were stated for addressing sedentary work. Organizations addressed sedentary work using a multi-faceted approach versus single approach and included numerous programs, practices, and policies at each level of the TWH HoC. These included:

- consultations focused on the principles of ergonomics applied to work environments,
- sit-stand workstations,
- time during work hours for physical activity,
- healthy movement policy,
- enhanced stairwells to encourage use,
- walking meetings,
- access to indoor and outdoor walking trails, and
- on-site and off-site recreation activities.

The redesign control was the most discussed control for addressing sedentary work. Redesigning the work environment to address sedentary work was often perceived as eliminating the working conditions that threaten the safety and health of workers and aligned with a work-related risk assessment. Organizations B and E discussed how they addressed sedentary work by redesigning the work environment with an underlying aim to reduce or eliminate work-related hazards.

“We took our corporate headquarters with 800 employees and built a new facility [and] significantly remodeled all spaces…There were opportunities to incorporate in our design process…using TWH principles. Our office furniture is standardized to electric sit-stand desks…Eliminating some of the working conditions for sedentary work was a significant risk for us [to address].”

[Organization B]

When organizations discussed their approach for addressing sedentary work, aspects of the inner setting domain were prominent. Constructs included implementation climate (i.e., culture) and readiness for implementation (i.e., available resources and leadership engagement). For example, Organizations G and F discussed how the culture of the organization supports their multi-level efforts for encouraging choices for healthy movement throughout the workday.

“[We have] things like indoor walking trails and efforts to make stairwells improved. Healthy movement policy is a lot of elements so the whole environment coaxes you to make the right choice…Culturally, from the CEO level down, being active and taking time to workout isn’t seen as evil…We’re putting our energies and having the cultural support to go in the right direction.”

[Organization G]

#### 3.3.4. Subtheme: Implementation Process Was a Likely Facilitator or Barrier for Organizational Efforts That Prevent Work-Related Stress

The eliminate control was not used as a control to address work-related stress in any of the organizations. The controls of substitution, redesign, educate, and encourage were among several controls reported to be used by organizations to address work-related stress. Organizations reported other strategies to address work-related stress that aligned with principles of a TWH approach, such as listening to workers to address their needs. Various programs, policies, and practices to address work-related stress included the use of an onsite psychologist, instruction of meditation and/or yoga, use of employer assistance programs, and leadership and worker resiliency training. Mental health promotion, resilience building, emotional intelligence, psychological safety, emotional well-being, and harassment and violence prevention were other terms used when asked to discuss approaches for addressing work-related stress. Often, the referenced controls that addressed work-related stress were concurrently used to address other work-related conditions, such as fatigue and sleep, productivity, and violence prevention.

Though organizations were at different stages of development for addressing work-related stress, respondents commonly referred to planning, engaging, and reflecting and evaluating (constructs of the implementation process) when discussing their approaches for addressing work-related stress. For example, Organizations A, E, F, and G talked about the use of external change agents to influence executive decisions on policy, organization-wide listening tours, individual risk assessments coupled with health education, and job-specific and company-wide surveys for planning organizational programs to address work-related stress.

“Regarding work-related stress, [we] use a validated tool to identify work groups to identify [stressors] and remove stress hazards from the workplace…They work to remove the stressors and eliminate them. When [it is] not possible to remove, they change them. Change the decision-making process, [change the] amount of work, decide how to do it in a different way, [or] redesign.”

[Organization F]

Relevant to the construct of engaging, Organizations C, D, F, and G discussed the use of training programs at the levels of leadership and workers for addressing work-related stress.

“Have training for employees and leaders because they need to understand stress differently. Especially leaders need to understand the role they have in causing or creating stress for workers.”

[Organization F]

### 3.4. Theme 4: Implementation Climate Primes Benefits and Obstacles

The inner setting domain was notable considering the experienced benefits, obstacles, and lessons learned reported by organizations using an approach consistent with a TWH approach. The implementation climate was a pervasive construct (of the inner setting domain) throughout the respondents’ discussions on benefits, obstacles, and lessons learned.

#### 3.4.1. An Existing Implementation Climate Supports the Benefits Experienced

Relevant implementation climate sub-constructs discussed for benefits experienced by organizations included relative priority, compatibility, and organizational rewards and incentives. In terms of relative priority (i.e., perceived importance of implementation), respondents discussed the TWH approach as a useful way to leverage plans that addressed worker well-being through their organizational safety strategy.

“Focus on how we stop killing people, then worry about wellness. Because of the credibility and foundation of [the TWH] approach, we incorporated wellness, and I would say that wellness programs are being developed in every single [unit] in the [organization]. I couldn’t have gotten this started. It helped going through the safety route to get wellness in.”

[Organization A]

Concerning compatibility, respondents referred to the TWH approach as a tool that provided a common language that could be understood across the entire organization.

“We’re decentralized. Having [a] national tool with common language was helpful. For me calling each [unit and saying], you need to do this wellness stuff wouldn’t work for everyone. People use different language for it.”

[Organization A]

Concerning organizational rewards and incentives, an organization’s demonstrated commitment to worker safety and health serves as a means for recruitment and retention.

“It’s helpful in recruitment as an employer of choice. [We] have people who apply for multiple jobs and work to get in the door. The culture with us being a mission-driven organization, we frequently hear the phrase we ‘walk the talk’. This is part of us doing that. [A] benefit [of the TWH approach] is contributing to positive culture. [We] have amazing longevity and retention with our employees.”

[Organization B]

#### 3.4.2. Subtheme: Lack of Implementation Climate and Absence of Readiness for Implementation Were Indicated as Obstacles

Implementation climate and readiness for implementation were notable constructs described by most organizations when discussing obstacles in implementing interventions relevant to TWH. Demonstrating the need for and importance of intervention implementation to leadership and to individual workers was prominently discussed as a barrier by most of the organizations. This is pertinent to leadership engagement (a sub-construct of readiness for implementation) and relative priority (a sub-construct of implementation climate construct). These constructs relate to leadership commitment for the implementation of the intervention and the shared perceptions of the importance of the intervention implementation within the organization. Organization C described challenges of first convincing workers to buy-in to the intervention.

“Naysayers. You could give a [worker] a gold bar, and they’ll complain about how heavy it is…Yoga was [seen as being] implemented passive aggressively at first with the mentality that you can go to yoga or [go to work]. You’ll take all the [work] calls [instead of yoga]. That was at first. Now they love it.”

[Organization C]

Organizations A and F described cautions to consider for preparing the implementation climate (a construct of inner setting domain) as it relates to engaging leadership and setting goals to ensure the long-term sustainability of the intervention.

“We set bold, audacious goals, but we’re mindful of not letting us go too far into every issue in the company. [You] have to be realistic and caution what’s really doable—making sure you think long-term but being realistic in what you can really do. Plan for what comes next.”

[Organization F]

Securing consistent leadership engagement for the intervention by demonstrating cost value of the investment in the long-term safety, health, and well-being of workforce was discussed as underlying obstacle among Organizations C, D, and G.

“We have the data, but operating centers don’t see [a] cost reduction. Executive team needs to know what we’re doing and [the] benefits of it. [The benefit] doesn’t always translate across leadership.”

[Organization D]

“[You] need to show broad value. It’s too simple to say, ‘I have an ROI.’ When we talk about recruitment, retention, and reputation, defining and measuring that is really hard. It’s a challenge and requires a lot of data management which is hard…Sometimes a challenge is understanding that it can’t be a 1–2-year investment. It can’t be a quick hit and get a benefit… [We have to] keep leaders reminded of [this time investment].”

[Organization G]

While Organization C recognized money and naysayers as obstacles for starting the intervention, they noted the importance of creativity at the early stages of intervention implementation.

“Don’t let money be a deciding factor in initial stages of developing and innovating. Money doesn’t exist at those initial conversations. Budgets are budgets. Don’t let money be a blocker.”

[Organization C]

#### 3.4.3. Subtheme: Organizational and Leadership Commitment Were Discussed as Lessons Learned for Successful Implementation of Organizational Interventions

Implementation climate and readiness for implementation were the most relevant inner setting domain constructs among all organizations when discussing lessons learned in developing organizational interventions consistent with a TWH approach. Leadership engagement (a sub-construct of readiness for implementation domain) was the most notably discussed construct among all the organizations. Despite leadership changes, Organizations E and F reported commitment for the implementation of their interventions was maintained through company-wide goals.

“One thing to consider is, to actually be able to implement TWH, it has to be a company priority. We are lucky because it’s a company 2025 goal. We have that frame. If we were just new to this initiative, it might not go as far. It has to be a company priority.”

[Organization F]

Amidst gaining leadership commitment and workers’ buy in, Organizations B and C, which had less mature programs, discussed an important underlying theme of tension for change (a construct of the inner setting domain) for getting their programs started.

“It was getting that leadership commitment. Don’t give up, it might take some time. Make sure employees are engaged and know what it’s all about.”

[Organization B]

Several organizations discussed the importance of aligning the intervention to a systems issue to gain the attention of leaders, or compatibility (a construct of the inner setting domain).

“By focusing on systems and environmental issues, [we avoid] the tendency to focus on folks at the individual intervention level versus restricting the café contract. I think that having that awareness and being prepared to look at it in that broader system, what are we doing as an organization? Clarifying that role is different from a traditional wellness program. That being effectively communicated is the biggest challenge to leadership support and employee buy-in.”

[Organization B]

Other inner setting domain constructs discussed as experiences gained from implementing an approach consistent with TWH included networks and communication, culture, goals and feedback, and relative priority.

“[You] can’t do it alone. That’s key. Partnering with stakeholders—it’s about creating synergies and getting people excited about a culture shift or approach. We’ve been focused on partnering and ensuring that we include our HR partners, [staff] leaders, and risk and safety [personnel]…Let’s learn from one another.”

[Organization E]

## 4. Discussion

This exploratory, qualitative study sought to investigate how the principles of the Hierarchy of Controls Applied to NIOSH Total Worker Health have been applied and implemented in select employment organizations in the USA, with a special focus on the work-related issues of fatigue and sleep, work-related stress, sedentary work, and tobacco control. Due to the nature of the study, these findings cannot be generalized beyond this small study population. However, this study provided experiential insight into how U.S. organizations are implementing an integrated approach for worker safety, health, and well-being during the study period. Using CFIR to conduct a thematic analysis of in-depth interviews with program implementers from widely varied organizations, four themes were identified that suggested potential facilitators and barriers for implementation of the TWH HoC or other related guidelines in an organizational context. 

These themes highlighted the potential significance of the inner setting (i.e., culture, implementation climate, and readiness for implementation) of organizations on the implementation of integrated worker safety, health, and well-being interventions. Additionally, the themes indicated that the characteristics of the interventions (i.e., intervention source, relative advantage, and adaptability) are important considerations for successful implementation of integrated work-related safety, health, and well-being interventions. Table 3 provides a summary of key results according to the levels of controls in the TWH HoC and work-related issues of special interest and highlights relevant themes. Overall, the practical significance of these findings draws from the need for an organizational culture that is supportive of Total Worker Health [27]. Further study using CFIR in its full context is needed to advance a more applied understanding of what factors influence the implementation of the TWH HoC among employment organizations.

These findings are consistent with Schult et al. [16] in that VA employees identified that organizational and structural elements effected employee safety, health, and well-being. The CFIR provided a useful framework for comprehensively examining organizational factors likely to influence the implementation of complex, integrated interventions that address worker safety, health, and well-being. Use of CFIR in the analysis of occupational safety and health innovations has been sparsely applied [6] and our study broadens the application of this framework to TWH policies, programs, and practices. Given the rapidly changing makeup of work, the workplace, and the workforce, these preliminary findings may serve as a building block for informing prospective studies and guidelines for designing programs, policies, and practices that align to the future of work.

Prior to this study, it was recognized that organizations are taking steps to apply principles of a TWH approach [12] and are implementing facets of the TWH HoC [15]. However, there was not a clear understanding of how organizations are implementing the TWH HoC or other related guidelines. These findings suggest that despite individual-level awareness of TWH-related guidelines, organizations of varying sizes and program maturity appeared prevention-focused, incorporated the tenets of TWH in their organizational efforts to address working conditions that threaten the safety, health, and well-being of their workforce, and sought to promote a culture of health in their workforce. These findings bear comparison of characteristics of the early adopter organizations including judicious innovation-decision, role models for other organizations, and communicate their subjective evaluation of the idea to their peer networks [25]. Further research is needed to understand the varying contexts of employment organizations and associate those contexts with characteristics of early adopter organizations.

Work-related issues of special interest in this study included fatigue and sleep, work-related stress, sedentary work, and tobacco control, as these topics were most requested from organizations seeking information from NIOSH. All participant organizations were applying multiple levels of controls to address these working conditions as well as several additional working conditions (i.e., workplace violence, access to healthy food, access to affordable healthcare, hazards related to work and workstation design). The participating organizations demonstrated numerous examples of how they individually and collectively applied all five levels of the TWH HoC to address potentially harmful conditions of work and work-related issues of concern.

In many instances, the participating organizations indicated they were applying multiple controls to address work-related issues (i.e., sedentary work, tobacco control, and sleep and fatigue) that threatened worker safety, health, and well-being, which is consistent with the Sorensen et al. [21] finding that implementation of integrated interventions occurs on a continuum. This is an intriguing early finding as it shows progressive use of methods by organizations, the complexities involved in implementing the TWH HoC in organizations, and that participating organizations did not demonstrate regression to the individual worker. Additionally, this finding suggests that the TWH HoC can serve as a model to guide decision-making related to the changing nature of work, changing workforce demographics, and the changing workplace. Tamers et al. [19] suggest a growing need for workers to concurrently manage the responsibilities present in their work and personal lives, and the TWH HoC model encourages a transdisciplinary approach for organizations faced with this consideration.

In their review of effectiveness of 38 TWH interventions, Anger et al. [15] found pairing of the levels of the TWH HoC and that pairing of the education and encourage controls were most common. The results of this study reinforced the idea that the description of the TWH HoC is not discreet and that further characterization of the TWH HoC is needed. Additionally, research is needed to explore and evaluate organizational determinants of implementing interventions that comprehensively address worker safety, health, and well-being.

One of the major lessons learned from this study is related to the open invitation for unlimited key informants for a time-limited organizational interview of one hour in length. In the interviews with three or less participants, all the interview questions were able to be thoroughly discussed and each participant was able to contribute equally. However, in hour-long interviews with more than three participants, the data collection was limited by time which did not permit the sharing of perspectives from all participants and more in-depth inquiry. As we consider future qualitative studies using semi-structured interviews with more than three key informants from an organization, the allotted time for the interviews would need to expand.

This study is limited by several factors. First, the sample size was small (*n* = 7), thus limiting the generalizability of the research. However, the small sample size provided an opportunity for a more thorough analysis of implementation of principles consistent with the TWH approach across seven organizations that varied widely according to industry, size, and program maturation. Second, because this is an exploratory study and data were limited to hour-long interviews, the entirety of each organization’s safety, health, and wellness or well-being programs was not investigated. Third, all five domains of the CFIR were not examined. Because the CFIR was used for analysis and not part of the data collection, only certain domains were relevant to this study. Fourth, the definitions for the TWH HoC are not discreet. In many instances, the respondents would provide examples of controls that often were characterized differently than the interviewers would characterize and are likely reflected in coding and interpretation of findings. Lastly, we did not collect information about possible gender and/or ethnic differences in the organizations studied. We have no information about how these differences may have impacted the outcome.

## 5. Conclusions

This exploratory study addresses an important gap in the field of TWH by providing a preliminary and practical understanding into how early adopter organizations of various sizes and sectors have applied principles consistent with the TWH approach. The study provided insights to assess and understand the potential strength of organizational factors that impact the implementation of integrated interventions that address worker safety, health, and well-being. These insights go beyond the knowledge gained from the more common approach of looking solely at whether TWH guidelines, such as the TWH HoC, are implemented. Use of the Consolidated Framework for Implementation Research to identify potential determining factors produced information on the facilitators, obstacles, and lessons learned by organizations during the implementation process.

This knowledge can be used in the future to develop more targeted guidance for employers as they seek to address worker safety and health and enhance worker well-being. Based on the knowledge gained from applying this novel methodology to the TWH approach, more rigorous, qualitative, and mixed methods research is needed to better understand how organizations adopt and implement interventions that promote and sustain worker well-being. Such understanding will become more urgent as work, the workplace, and the workforce rapidly evolve into a new, unknown future.

## Figures and Tables

**Table 1:** Characteristics of the Selected Promising Practice Organizations.

Organization Code	Organization Size	Sector	Span of Operations	Manufacturing or Non-Manufacturing	Program Maturity	No. Key Informants Interviewed
A	>500	Government	National	Non-manufacturing	<15 years	1
B	>500	Private	State/Regional/Local	Non-manufacturing	<15 years	2
C	<500	Government	State/Regional/Local	Non-manufacturing	<15 years	1
D	>500	Private	National	Manufacturing and Non-manufacturing	>15 years	2
E	>500	Private	State/Regional/Local	Non-manufacturing	<15 years	1
F	>500	Private	Global	Manufacturing	<15 years	4
G	>500	Private	Global	Manufacturing	>15 years	2
H	>500	Private	Global	Manufacturing	Unknown	N/A

**Table 2:** Description of Themes and Subthemes Related to Implementation of the Hierarchy of Controls Applied to NIOSH Total Worker Health among Selected Organizations.

**Theme 1: Recognition of the TWH Approach and the TWH HoC**				
Relevant CFIR Domains: Intervention Characteristics and Inner Setting				
There was varied awareness of the TWH approach and the TWH HoC among selected Promising Practice organizations.		The principles of TWH are part of existing organizational values for building a healthy work culture.		The TWH approach leverages with traditional occupational safety and health approaches to address worker health more broadly.
**Theme 2: Implementation of the TWH HoC**				
Relevant CFIR Domains: Intervention Characteristics and Inner Setting				
The Eliminate Control was commonly used and trialed.	Adaptability and resources are important in the Substitution of unhealthy working conditions.	The Redesign Control was the most frequently used control and it was likely to provide both quality and advantage.	The Education Control offers advantage by coupling with other organizational efforts.	An organizational culture built around healthier choice making underscores the Encourage Control.
**Theme 3: Barriers and Facilitators in Addressing Specific Work-Related Issues**				
Relevant CFIR Domains: Inner Setting and Implementation Process				
Leadership engagement, available resources, and access to information are possible facilitators or barriers for organizational efforts that focus on Work-related Fatigue and Sleep.		Culture and available resources were indicated as important supports for organizational Tobacco Control efforts.	Organizational culture and available resources were suggested to provide multi-level efforts for addressing Sedentary Work.	Implementation process was a likely facilitator or barrier for organizational efforts that prevent Work-related Stress.
**Theme 4: Implementation Climate Primes Benefits, Obstacles, and Lessons Learned**				
Relevant CFIR Domain: Inner Setting				
An existing implementation climate supports the Benefits experienced.		Lack of implementation climate and absence of readiness for implementation were indicated as Obstacles.		Organizational and leadership commitment discussed as Lessons Learned for successful implementation of organizational interventions.

**Table 3:** Summary of Key Results According to Levels of Controls in the Hierarchy of Controls Applied to NIOSH *Total Worker Health*^®^ and Work-related Issues of Special Interest.

Level of TWH HoC	Examples of Implementation in Organizations	Relevant Themes
Eliminate	Implementing company-wide tobacco-free policies, use of robots in material handling to eliminate occupational exposures to lifting heavy loads and use of awkward postures, elimination of sugar-sweetened beverages sold at the workplace, elimination of electric cords from floors to remove hazards for slips, trips, and falls, reductions in shiftwork rotations and hours to prevent work-related fatigue, and, use of machine guarding to prevent traumatic injuries.	Most commonly used and trialed control amongst study popuation Use of stepwise process, involving multi-levels of controls leading to eliminating and reducing working conditions that threaten the safety, health, and well-being of workers Emphasis on cultural perspective that concentrates on eliminating work conditions that could be threatening to employee safety and health, the first level of control
Redesign	A worker-tailored pacing program for production, health insurance based on salary, enhanced lighting for an aging workforce, upgraded facility for violence protection, and an onsite nap room for workers to re-energize during the workday or de-stress before leaving work.	Most frequently used control Use of redesign control focused on relative advantage and evidence strength and quality
Substitute	Healthy movement policies, access to an onsite psychologist, placement of healthier choices in workplace cafeteria, and use of a lift-assist device.	Success of the substitute control is relevant to the adaptability of the intervention and dedicated resources
Educate	Training for supervisors and leaders that aims to reduce work-related stress among workers, health risk appraisals accompanied by health education, tobacco cessation training, corporate athlete programs, and onsite teaching of yoga and mindfulness meditation retreats for workers.	Offers an advantage by coupling with other organizational efforts Second most frequently used control
Encourage	Employee recreational equipment to use during breaktime and meetings, special interest groups and outdoor clubs, encouraging civility in the workplace, health coaching offered through employee assistance programs (EAP), and periodic health-related workplace campaigns.	Underscored by organizational culture built around healthier choice making Culture, implementation climate, and readiness for implementation are likely key determinants
**Work-related Issue of Special Interest**		
Sleep and Fatigue	Limited work hours during peak seasons, access to onsite nap programs and quiet rooms, engaging leadership in encouraging frequent rest and stretch breaks, peer support, worker training and education, providing flexible work schedules, and offering sleep care benefits through healthcare plans.	Leadership engagement, available resources, and access to information are possible facilitators or barriers Redesign, education, and encourage controls were most commonly used
Sedentary Work	Consultations focused on the principles of ergonomics applied to work environments, sit-stand workstations, time during work hours for physical activity, healthy movement policy, enhanced stairwells to encourage use, walking meetings, access to indoor and outdoor walking trails, and on-site and off-site recreation activities.	Organizations addressed using a multi-faceted approach versus single approach Redesign control was the most discussed
Tobacco Control	Organizational-wide tobacco-free policies, policies that emphasize tobacco free requirements on all property, including vehicles, prohibited use of electronic cigarettes, and education and encourage tobacco cessation at work and away from work	Culture and available resources were indicated as important supports for organizational tobacco control efforts Organizations commonly use of multi-level controls
Work-related Stress	Listening to workers to address their needs, use of an onsite psychologist, instruction of meditation and/or yoga, use of employer assistance programs, and leadership and worker resiliency training.	Implementation process a likely facilitator or barrier for organizational prevention efforts

## Data Availability

The data presented in this study are available on request from the corresponding author. The data are not publicly available due to privacy reasons.

## Appendix A

**Table A1:** Descriptions of Consolidated Framework for Implementation Research (CFIR) Domains and Constructs Applied to Total Worker Health.

Domain Primary Contextual Factors That Influence Implementation Effectiveness	Constructs Meta-Theoretical Factors That Help to Explicate Implementation	Application to Study
Intervention Characteristics	Intervention Source	Perception by key implementation leaders that the intervention (TWH HoC or TWH-related approach) was internally or externally developed. Relates to awareness of TWH.
	Evidence Strength & Quality	Perceptions of implementers of the efficacy of TWH-related interventions on improved worker safety and health outcomes and organizational outcomes.
	Relative Advantage	The alternative is not applying an integrated TWH approach or TWH HoC. This may vary according to OSH issue and/or organization.
	Adaptability	TWH HoC or TWH-related approach can be adapted or refined to meet the safety, health and well-being needs of workers and the organizational priorities.
	Trialability	The ability of the TWH HoC or TWH-related approach to be piloted or de-implemented if warranted.
	Complexity	Perceived difficulty of implementing TWH HoC by the key implementation leaders. Interview Question
	Design Quality and Packaging	Perceived excellence by key implementation leaders in how the TWH HoC or TWH-related approach is packaged.
	Costs	Costs associated with implementing the TWH Hoc or TWH-related approaches in the organization including investment, supply, and opportunity costs.
Outer Setting	Worker Community Needs & Resources	The extent to which worker’s community and family’s needs, as well as barriers and facilitators to meet those needs, are accurately known and prioritized for the organization.
	Cosmopolitanism	The degree to which the organizations are networked with other external organizations
	Peer Pressure	Competitive pressure to implement TWH HoC or TWH-related approaches because most other key peer organizations have already implemented/competitive edge.
	External Policy & Incentives	Other national recommendations, policies, or reporting benchmarks that address the safety, health, and well-being of workers (i.e., OSH VPP program)
Inner Setting	Structural Characteristics	The social architecture, age, maturity, and size of an organization. Part of the demographic characteristics collected in the sampling
	Networks & Communication	The nature and quality of webs of social networks and the nature and quality of formal and informal communications within an organization. This relates to the interactions and integration among different organizational units responsible for the safety, health, and well-being of its workforce.
	Culture	Norms, values, and basic assumptions of a given organization—especially in relation to the worker safety and health culture.
	Implementation Climate	The absorptive capacity for change, shared receptivity of involved individuals to TWH-related interventions or the TWH HoC, and the extent to which use of TWH-related approaches will be rewarded, supported, and expected within their organization.
	*Tension for Change*	The degree to which the key implementers believe the current situation as intolerable or in need of change.
	*Compatibility*	The degree of tangible fit between meaning and values attached to the TWH HoC or TWH related approaches by key implementers, how those align with individuals’ own norms, values, and perceived risks and needs, and how the TWH HoC or TWH-related approaches fits with existing workflows and systems
	*Relative Priority*	Key implementers shared perception of the importance of the TWH HoC or TWH-related approach within the organization.
	*Organizational Incentives & Rewards*	Extrinsic incentives such as goal-sharing awards, performance reviews, promotions, and raises in salary, and less tangible incentives such as increased stature or respect.
	*Goals and Feedback*	The degree to which goals are clearly communicated, acted upon, and fed back to staff, and alignment of that feedback with goals.
	*Learning Climate*	A climate in which: (a) leaders express their own fallibility and need for team members’ assistance and input; (b) team members feel that they are essential, valued, and knowledgeable partners in the change process; (c) individuals feel psychologically safe to try new methods; and (d) there is sufficient time and space for reflective thinking and evaluation.
	Readiness for Implementation	Tangible and immediate indicators of organizational commitment to its decision to implement the TWH HoC or TWH-related approach.
	*Leadership Engagement*	Commitment, involvement, and accountability of leaders and managers with the implementation.
	*Available Resources*	The level of resources dedicated for implementation and on-going operations, including money, training, education, physical space, and time.
	*Access to Knowledge Information*	Ease of access to digestible information and knowledge about the TWH HoC or TWH-relate intervention and how to incorporate it into work tasks.
Characteristics of Individuals	Knowledge & Beliefs about Intervention	Key implementation leader’s attitude toward and valued placed on the TWH HoC, as well as familiarity with facts, truths and principles related to the intervention.
	Self-efficacy	Key implementers belief in their own capability to execute course of action (i.e., TWH HoC) to achieve implementation goals (i.e., improved worker safety, health and well-being and organizational outcomes).
	Individual Stage of Change	Key implementer’s characterization of the phase they are in the implementation of the HoC or TWH-related intervention.
	Individual Identification with Organization	Describes how the key implementers perceive the organization and their relationship and degree commitment with that organization.
	Other Personal Attributes	Personal traits of the key implementer (i.e., competence, capacity, motivation, values, tolerance of ambiguity)
Implementation Process	Planning	The degree and quality to which the organization has developed an approach to implement the TWH HoC or related intervention
	Engaging	The degree to which the organization has appointed people to lead the implementation of the TWH HoC or related approach and use a combined strategy to implement in the organization (i.e., social marketing, training, role modeling, education).
	*Opinion Leaders*	Individuals who have formal or informal influence on the attitudes and beliefs of their colleagues with respect to implementing the TWH HoC or TWH-related intervention
	*Formally Appointed Opinion Leaders*	Individuals from within the organization who have been formally appointed with responsibility for implementing an intervention as coordinator, project manager, team leader, or another similar role.
	*Champions*	Individuals who dedicate themselves to supporting, marketing, and driving through an implementation.
	*External Change Agents*	Individuals who are affiliated with an outside entity who formally influence or facilitate intervention decisions in a desirable direction
	Executing	The degree to which the organization’s TWH-related intervention was carried out according to plan.
	Reflecting and Evaluating	Feedback (qualitative and quantitative) about the progress, experience, and quality of implementing the TWH HoC or TWH-related approach. This relates to an interview question.
